# Greenhouse gas emissions as a result of spectators travelling to football in England

**DOI:** 10.1038/s41598-017-06141-y

**Published:** 2017-08-01

**Authors:** Adekunle Dosumu, Ian Colbeck, Rachel Bragg

**Affiliations:** School of Biological Sciences, University of Essex, Wivenhoe Park, Colchester, CO4 3SQ England

## Abstract

Transport remains a critical avenue in the attempt to reduce greenhouse gas (GHG) emissions and any significant effort to reduce travel GHG emissions will need to encourage a movement towards more fuel-efficient, less polluting behaviours. The aim of this paper is to calculate GHG emissions arising from the travel of spectators to and from football games within eight football tiers (3 to 10) in England, and to extrapolate this to a national level. The study comprised of 1649 participants with an average age of 42 years (*M* = 42.63, *SD* = 17.10). Participants travelled to and from games by walking, cycling, car, bus, train or taxi. The average distance travelled to and from games was 41.5 km. A Kruskal-Wallis test was conducted to evaluate differences in travel related GHG emissions between the eight football tiers during the 2012/13 season. The results indicate significant differences between football tiers’ GHG emissions, H(7) = 46.474, *p* < 0.001. The annual GHG emission of spectators from the 8 tiers for the 2012/13 season was estimated at 56,237 tonnes of CO_2_e, accounting for less than 0.05% of transport emissions in England. Football authorities should have robust travel plans and educate spectators to employ more sustainable travel plans to games.

## Introduction

The 2014 Intergovernmental Panel on Climate Change report notes that greenhouse gas emissions have increased over the last decade and are now growing at almost twice the previous rate^1^. International agreement has been reached with the intention of limiting global warning to 2 °C^2^. In 2010, the transport sector globally produced 6.7 GtCO_2_ of direct greenhouse gas (GHG) emissions^1^, and in Europe, road transport contributed about 20% of the European Union’s (EU) total GHG emissions. This figure has increased by nearly 23% between 1990 and 2010. Moreover, transport is one of the major sectors in the EU where GHG emissions are still on the increase^3^ and within the UK its contribution has increased from 16% in 1995 to 24% in 2015^4^. Cars account for over 58% of these emissions followed by heavy goods vehicles (15.8%), light vans (14.6%), buses 3.2% and trains (1.7%).

Transport remains a critical avenue in the attempt to reduce GHG emissions, and any significant effort to reduce emissions will need to encourage a movement towards more fuel-efficient, less GHG emitting transport behaviour. Dolf and Teehan^5^ suggested that the link between climate change and travel to sporting events had implications for how participants travelled to them. In England, football is a popular national sport which is spectator dominated. In 2012, over 60 million spectators from tiers 1 to 11 watched football in England^6, 7^. The study by Campaign for Better Transport in 2013 showed a dependence on the use of cars to travel to games and reported limited car sharing behaviour among the fans^8^. The environmental impact of travel to football is starting to be recognised and addressed; the Premier League and Football Association (FA) are now encouraging clubs to consider the environmental impacts of their operations and take action to minimize those impacts^9^. Currently, those engaged with football are increasingly starting to make it sustainable: economically, socially and environmentally^10^.

For instance, the FIFA World Cup in Germany in 2006 generated approximately 250,000 tonnes of greenhouse gases^11^, while the World Cup in South Africa, in 2010, generated 1.65 million tonnes of CO_2_e^12^. Within the UK Collins and co-workers^9, 13^ calculated the ecological footprint of the 2004 FA Cup Final. In 2007 they also undertook a review of the environmental impacts of the operations of clubs in the Premiership League based on literature on individual club websites. Of the 20 clubs reviewed, few were involved in projects to minimize their environmental impacts^9^. In 2006 Ipswich Town claimed to be the UK’s first carbon neutral club^14^ although only 3,000 fans out of a fan base of well over 20,000 participated in the initiative.

With the recent development of corporate social responsibility (CSR) initiatives by football clubs many premier league clubs have now gone beyond compliance with environmental rules and regulations^15^. Championship clubs have also embraced ambitious sustainability strategies. For example Newcastle United have installed a combined heat and power system at its St James Park Stadium^16^ while Bristol City have a new solar PV project at its Ashton Gate Stadium^17^. Gibbons and Nuttall^18^ have noted that lower echelons of English football have largely been ignored by such globalization pressures.

Apart from studies on GHG emissions from mega sporting events, limited research has been carried out on GHG emissions from smaller scale football matches, such as league games in England, and none have considered GHG emissions as a result of spectators across the lower league levels. Spectator travel to matches is a significant aspect of the sport and it should be seen from a sustainable perspective because travel and sustainability are both linked, although it can often be interpreted to mean minimizing the amount of car travel and thereby reducing the amount of GHG emissions. However, attention should also be given to transport policies because of their environmental outcomes without excluding economic and social consequences as these occur at different scales^19^.

The football system in England is made up of 11 tiers, consisting of Premier League, Championship, League One and League Two with 92 clubs. The Non-League comprises of seven tiers: National League (formerly Football Conference Premier Division) down to County Leagues with over 1,100 clubs (20 clubs in tier 1 and 338 clubs in tier 10). The lower tiers (from tier 5 to 11) cover smaller geographical areas with fewer spectators compared to the upper tiers (tier 1 to 4). When aggregated nationally, however, the number of spectators at non-league football is significant when compared with the league system. The aim of this paper was to calculate travel GHG emissions for football clubs within the lower eight tiers, from tier 3 to tier 10 in England and to extrapolate this to a national level.

## Methodology

In collaboration with Essex Football Association, 47 clubs were selected from 8 football tiers in Essex. These clubs were selected to reflect a broad representation of football spectators across the football tiers in England (Table 1). A questionnaire (Supplementary Information) was used to examine travel GHG emissions of football spectators and extrapolated to national level.

**Table 1:** Number of participating clubs from 8 football tiers in England.

Football Tier	League Name	Number of Clubs
3	League One	2
4	League Two	4
5	Conference Premier	2
6	Conference South	4
7	Isthmian Premier	6
8	Isthmian Division 1 North	12
9	Thurlow Nunn Premier Division	6
10	Essex Senior League	11
Total		47

The study was conducted from mid-February 2012 to March 2013. In order to determine the travel related GHG emissions from spectators, a questionnaire-based study was employed, using both hardcopy and online questionnaires to collect data. The questionnaire incorporated questions on: demography, travel details and attendance frequency. All respondents provided informed written consent before completing the questionnaire. The study was approved by the University of Essex, Faculty of Science and Health Ethics Committee and conducted in accordance with relevant guidelines and regulations.

The GHG emissions were calculated using the distance travelled to and from games, mode of travel and Defra’s greenhouse gas conversion factors^20^. The greenhouse gas emissions calculated included CO_2_, CH_4_ and N_2_O. The values for CH_4_ and N_2_O are presented as CO_2_ equivalent (CO_2_e) using Global Warming Potential factors^21^.

Defra conversion factors for 2012 were used to calculate GHG emissions by multiplying the data on the distance travelled to and from games by the appropriate conversion factor as shown in Table 2.

**Table 2:** Defra’s GHG emission conversion factors 2012.

Mode of transport	Defra’s Conversion factor (kgCO_2_e)	Remarks
Walking	0	No fuel
Cycling	0	No fuel
Car (unknown fuel)	0.19	kgCO_2_e/km/car
Bus (Average local)	0.11	kgCO_2_e/km/person
Train (National Rail)	0.06	kgCO_2_e/km/person
Taxi (Regular)	0.23	kgCO_2_e/km/person

Note: The distance travelled by participants was measured in miles but converted to kilometres (1 mile = 1.61 km). The emission per spectator was calculated by multiplying distance travelled to and from games by appropriate conversion factor based on mode of travel and reported in kgCO_2_e. For example 10 km travel to and from a football game by bus will result in 10 * 0.11 = 1.1 kgCO_2_e^20^.

A power analysis was performed using GPower 3.1 software to calculate the recommended sample size for this study. Based on a one-way ANOVA test with 8 groups, medium effect size and 95% power, a total sample size of 360 was recommended. The achieved sample size of 1,649 has 100% statistical power to detect all significant effects that may exist in the study data. Significance was set as a *p*-value of <0.05.

Data was analyzed with IBM SPSS version 20. The test for normality for the outcome variable (GHG emissions) was not met, which led to using non-parametric tests on the data. The statistical tests employed were: descriptive statistics, Kruskal-Wallis test, and Mann Whitney U tests.

## Results

1,649 football spectators completed the questionnaire. The participants were 1,315 men (80%) and 334 women (20%), with an age range of 18 to 84 years, and mean age of 42 years (*M* = 42.63, *SD* = 17.10).

The distance travelled to and from games ranged widely from 80 metres to 612 km, with the mean distance travelled of 41.5 km to the games (*SD* = 72.89) as shown in Table 3. The mean distance travelled to and from home games was 15.55 km (*SD* = 24.29) while the mean distance travelled to away games was 114.12 km, (*SD* = 106.57). The spectators at the non-league level (tiers 5 to 10) travelled less distance to games (*M* = 25.15 km, *SD* = 46.22) than spectators at the league (tiers 3 and 4) level (*M* = 62.82 km, *SD* = 92.94). The distance travelled to and from games varied across the football tiers, home and away games as shown in Table 3.

**Table 3:** Mean distance travelled by home and away spectators at 8 football tiers.

Football Tier	Football tier		Home		Away	
	Mean (km)	Std. Deviation	Mean (km)	Std. Deviation	Mean (km)	Std. Deviation
3	66.92	101.65	20.21	26.05	231.69	97.71
4	59.40	85.01	13.46	24.58	177.17	70.05
5	51.35	110.23	21.92	32.34	190.10	208.53
6	27.12	39.33	17.31	29.76	51.24	49.03
7	26.48	34.62	10.55	15.21	68.84	36.19
8	22.41	37.86	16.47	20.32	51.47	74.36
9	19.12	31.42	11.63	22.67	53.50	42.19
10	18.40	23.36	13.65	24.06	23.68	21.49

**Figure 1:**
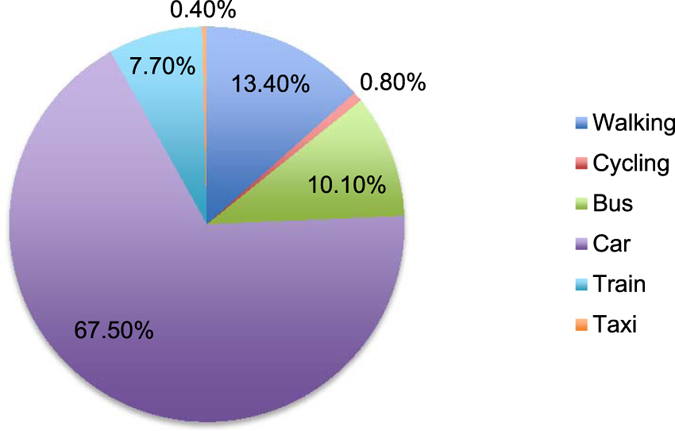
Participants’ percentage mode of travel across the football tiers.

The most frequent mode of travel by the participants to and from games was by car (67.5%, n = 1,113), followed by walking (13.4%, *n* = 221), then bus (10.1%, *n* = 166), train (7.7%, *n* = 127), cycling (.8%, *n* = 14) and taxi (.4%, *n* = 7) as shown in Fig. 1. The number of people in each car ranged from 1 to 7, with an average of two persons travelling in each car (*M* = 2.26, *SD* = 1.12).

### GHG emission among the 8 football tiers

GHG emissions were estimated based on the assumption from distance travelled by each spectator using UK average emission factors for different modes of travel and are presented per passenger, rather than per vehicle^20^. The mean GHG emission among the participants was 4.74 kgCO_2_e (*SD* = 9.48, range: 0–91.04 kgCO_2_e)_._ The GHG emission data were highly skewed and could not be used for parametric analyses. The mean GHG emission from home spectators was 1.70 kgCO_2_e (*SD* = 2.89) and that of away spectators 13.71 kgCO_2_e (*SD* = 14.95). The spectators at the non-league level emitted less travel related CO_2_ (*M* = 2.81 kgCO_2_e, *SD* = 5.90) than spectators at the league level (*M* = 7.24 kgCO_2_e, *SD* = 12.26).

**Figure 2:**
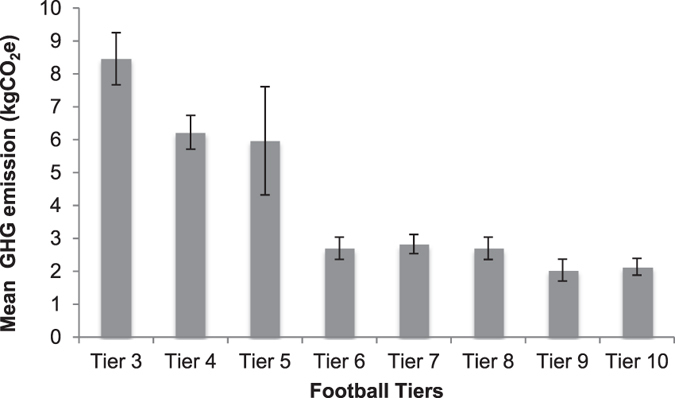
Mean GHG emissions per spectator across eight football tiers (N = 1,406). Error bars denote mean ± standard error of the mean.

A Kruskal-Wallis test was conducted to evaluate differences in GHG emissions between the eight football tiers in England from travel during the 2012/13 season. The results indicate significant differences between football tiers’ GHG emissions, H(7) = 46.474, p < 0.001. Further pairwise comparison testing with adjusted p-values showed that there was a significant difference in GHG emissions which were lower in tier 8 compared with tier 3 (p < 0.05). Additionally, spectators in tier 8 had significantly lower GHG emissions compared with spectators in tiers 3, 4, 5, 6 and 7 (p < 0.05). Finally, the spectators in tier 10 had significantly lower GHG emissions than spectators in the tiers 3, 4, and 5 (p < 0.05). Figure 2 further illustrates the differences in the GHG emissions among the 8 football tiers.

**Figure 3:**
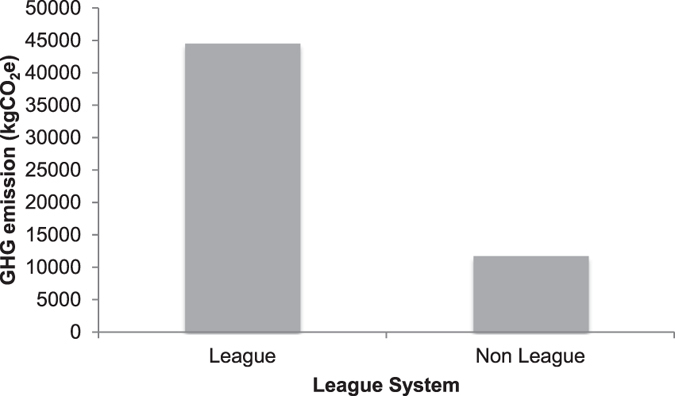
Travel related GHG emissions between league and non-league clubs in England.

The GHG emissions from the league level (tiers 3 and 4) were estimated to be 44,473.30 tonnes of CO_2_e while the non-league levels emitted 11,764.09 tonnes of CO_2_e, suggesting that the GHG emission at the league level was about four times more than the non-league level as shown in Fig. 3.

A Mann Whitney U test was conducted to compare the GHG emissions between two groups of participants: home and away. The result revealed a statistically significant difference with higher GHG emissions from away (N = 417) fans M = 13.71 (SD = 14.95) rather than home (N = 1232) fans M = 1.70 (SD = 2.89), U = 87371.50, *z* = −20.766, *p* < 0.001, r = 0.51-large effect size.

### National extrapolation of GHG emissions of participants

The annual number of spectators and mean GHG emission at each football tier were used to linearly extrapolate^22^ the total travel GHG impact of spectators for the 2012/13-football season, resulting in a total travel emission of 56,237.39 tonnes of CO_2_e, as shown in Table 4 (i.e. mean GHG emissions at various tiers multiplied by annual number of spectators at that tier). This extrapolation is based on the assumption that clubs in this study are representative of clubs across England. This is a reasonable first approximation although it is possible that some leagues in the lower tiers (e.g. North West Counties Premier Division) may have more clubs in a large metropolitan region making access easier to public transport.

**Table 4:** National Extrapolation of GHG emissions for 2012/13 football season.

Football Tier	2012/13 season annual attendance	Mean GHG emission (kgCO_2_e)	Mean GHG emission (tonnes of CO_2_e)
3	3,473,154	8.46	29,382.88
4	2,422,218	6.23	15,090.42
5	1,041,886	5.97	6,220.06
6	539,217	2.69	1,450.49
7	413,765	2.83	1,170.95
8	463,398	2.69	1,246.54
9	539,959	2.04	1,101.52
10	268,470	2.14	574.53
Total	**9,162,067**		**56,237.39**

Source: The annual attendance at football tiers^7, 23^, National emissions obtained by multiplying annual attendance at each tier by respective mean GHG emissions.

## Discussion

The aim of this paper was to calculate GHG emissions arising from the travel of spectators to and from football games within the lower echelons of the football leagues in England (tiers 3 to 10), and to extrapolate this to a national level. This research did not cover emissions from energy consumption, food and drinks consumed at games and emissions from spectator waste sent to landfill. This study revealed that the football spectators in England are predominantly males (80%). This is similar to findings from studies from other countries^24–26^. Findings from this study show that travelling to and from football games can have a significant impact on the environment, with the majority (67%) of spectators using cars and average travel emissions from this study were 4.74kgCO_2_e. The reliance on cars can be attributed to time savings and convenience. The average occupancy rate of cars (M = 2.26) was similar to that reported for sporting events in the US with 2.47^27^ and 2.7 in Canada^5^.

By analysing data form 3,474 adults in the UK the transport related GHG emissions for a number of activities (commuting, business, shopping, and social/leisure) have been calculated^28^. The mean carbon emissions per person per week were reported to be equal to 35.1 kgCO_2_. Overall commuting accounted for 35% of the weekly emissions followed by social trips (24%), business trips (19%) and travel for shopping or personal business (19%). Based on these figures attendance at football matches would make a significant contribution to the weekly emissions from social trips.

Also, this study demonstrates that GHG emissions significantly differ across the eight football tiers. Tier 4 had significantly higher GHG emissions than tier 9; GHG emissions were higher in tier 3 than in tiers 7, 8 and 10. The league system emitted almost four times more GHG than the non-league teams; this is a result of both greater distances between clubs and more overall spectators. GHG emissions from travelling to away games was more than the GHG emissions from travelling to home games, this is similar to the findings from another study^29^.

In 2012, the total GHG emission in the UK was 474.1 (MtCO_2_e), with 116.9 (MtCO_2_e) from the transport sector^30^. This study showed that the extrapolated national GHG emissions of football spectators was 56,237.39 (kgCO_2_e) during the 2012/13 football season from tiers 3 to 10 and this accounted for less than 0.05% of the annual GHG emission from the transport sector nationally in 2012. Findings from this study show travel contributed to GHG emissions particularly from the use of cars and public transport. This is similar to study for the FA Cup tournament for the whole of 2007/8 which generated 42,000 tonnes CO_2_e from 731 teams^31^. Attendances for the 2016/17 season show an average increase of 18% on that used in this study (2012/13). More people are choosing to follow their local clubs and so although small, the contribution of spectators to GHG emissions is increasing.

Cox^32^ argues that in the Premier League the distance between stadia is linked to the number of spectators. Overall the average distance is 141 miles. As distances increase attendance decreases which reflects rivalry between local teams and increased traveling costs. Distances covered in Leagues One and Two, as well as the National League are greater than in the Premier League. The lower league clubs are often based in small towns and so may well be less well provided for by public transport thereby increasing the use of cars. Hence the means of transport and corresponding emissions, traffic density and distances among the competing clubs at the different football tiers are not uniformly distributed across the country.

The findings of the research by the Campaign for Better Transport (CfBT) in 2013 are similar to this study. Both studies found that cycling to games by spectators was rare and that car sharing was higher for away travel. Both studies found that home spectators in the lower tiers were more dependent on the car (40%, 45%, 58%, 51% in the Premier League, Championship, League 1 and League 2 respectively) as there are fewer alternatives to the car. However, the primary difference between both studies was that the main mode of travel from the study of CfBT was the train. Given that that the CfBT study^8^ involved both Premier and Championship teams and didn’t consider teams in tier 5 and below this is not surprising. However it did note that transport costs account for approximately 25% of Premier League fans’ expenses on match day whereas for supporters of League Two clubs this figure increased to 33%.

The Scottish FA, Scottish Premier League and Scottish Football League undertook a National Football Survey in 2013^33^. Here it was found that 64% of spectators travelled to matches by car; similar to our figure of 67%. A quarter of supporters walk to the stadium which suggests that a large number of spectators support their local club. However excluding a ticket for the game travel to/from the match was the most significant match day cost. Similar concerns were identified in the CfBT study^8^. The figure of 13% of spectators walking to games found in our survey is much higher than at many other sporting events^5^.

It should also be noted that in the higher echelons of football sports tourism is increasing in popularity. In 2014 800,000 visitors to the UK went to a football match^34^ with Manchester United, Arsenal and Liverpool receiving approximately 100,000 each. Such visitors have a significant impact on the GHG emissions due to travel. In British Columbia Dolf and Teehan^5^ found that although 4% of spectators travelled by air, this contributed 52% of travel related GHG emissions.

Despite the fact that football has a negative environmental impact, particularly from spectators travelling to and from games, a behavioural change in travel plans from individuals can have a great benefit in controlling GHG emissions, possibly by an increase in vehicle occupancy rates, or ideally by increased low-emission travel mode choices.

Within the German Bundesliga clubs have introduced a combined ticket which provides entry to the stadium as well as for the free use of public transport on match days. Such a system was first offered by FC Cologne in 1983 and is now offered by the majority of Bundesliga and lower leagues clubs^35^. At the start of the 2015/16 season the Football League commenced an initiative with Liftshare.com whereby fans post details of their match day travel and offer their spare car seats to other fans. As yet there are no details as to how successful this has been. Previous attempts, such National Express’s partnership with the FA, to provide solutions to reduce the carbon impact of fans’ travel have fallen by the wayside.

Further afield Reiche^36^ notes that some clubs in the US National Football League encourage the use of electric cars by providing fans with charging stations while some of the richer teams offset the emissions from away-game travel, for example by tree planting initiatives.

Research into sports tourism has found that individuals tend to engage less in pro-environmental behaviours in a tourism context compared to their behaviour at home^37^. There is currently little to suggest that this applies to away spectators. However improved awareness of a destination’s environmental responsibility prior to the match may have an impact on their environmental behaviour at the destination.

Comparing this study to previous studies attests to the fact that a more environmentally friendly way of travel by spectators will lower GHG emission and could have a positive impact on the climate^38–40^.

## Conclusion

This study has compared the GHG emissions of eight football tiers in England during the 2012/13-football season and extrapolated those emissions to the national level. The annual GHG emissions of football spectators from the 8 football tiers were estimated at 56,237 tonnes of CO_2_e accounting for less than 0.05% of transport emissions in England. Tiers 3 and 4 had significantly higher GHG emissions compared to tiers 7, 8, 9 and 10. It should be noted that in the upper football league tiers emissions are produced, not only as teams and fans travel to matches, but as stadiums are built, run and maintained. In the lower tiers virtually all emissions are transport related.

This study highlights the different travel regimes for spectators at the lower tiers of English football compared to those in the Premier League. An improved understanding of travel to such games is important because although they may have small individual carbon footprints, they happen in much larger numbers.

This study has identified a clear environmental problem caused by spectators travelling to games even on a national scale. Football authorities should have robust travel plans and educate spectators to employ more sustainable travel plans to games. Both an individual and national approach could be employed to reduce the GHG emission from spectators, and the Department for Transport (DfT) can take the lead by liaising with all the parties involved in football, from the local level to the national level, by introducing a “Kombi Ticket” system similar to that operating in Germany so that match tickets can be used consistently and universally for local and regional travel to matches^35^. Creating long-term use of public transport is something that football clubs should attempt to achieve.

## Electronic supplementary material

Supplementary Information
