# Supplementary material for: Greenhouse gas emissions as a result of spectators travelling to football in England
Source: Sci Rep. 2017 Aug 1;7:6986. doi: 10.1038/s41598-017-06141-y (PMC5539281; doi:10.1038/s41598-017-06141-y)
Supplement: Supplementary file 1 — Supplementary Information [file 41598_2017_6141_MOESM1_ESM.doc]

Greenhouse gas emissions as a result of spectators travelling to football in England

Adekunle Dosumu, Ian Colbeck* and Rachel Bragg

School of Biological Sciences, University of Essex, Wivenhoe Park, Colchester CO4 3SQ

*Corresponding author: E-mail [colbi@essex.ac.uk](mailto:colbi@essex.ac.uk)

Supplementary information: Questionnaire on travel GHG emissions of Football Spectators in England
